# Re-characterization of the Glomerulopathy in CD2AP Deficient Mice by High-Resolution Helium Ion Scanning Microscopy

**DOI:** 10.1038/s41598-017-08304-3

**Published:** 2017-08-16

**Authors:** Kenji Tsuji, Teodor G. Păunescu, Hani Suleiman, Dongping Xie, Fahmy A. Mamuya, Jeffrey H. Miner, Hua A. Jenny Lu

**Affiliations:** 1 Center for Systems Biology, Program in Membrane Biology and Division of Nephrology, Department of Medicine, Massachusetts General Hospital, and Harvard Medical School, Boston, MA USA; 2 Department of Pathology and Immunology, Washington University School of Medicine, St. Louis, MO USA; 3 Division of Nephrology, Department of Medicine, Washington University School of Medicine, St. Louis, MO USA

## Abstract

Helium ion scanning microscopy (HIM) is a novel technology that directly visualizes the cell surface ultrastructure without surface coating. Despite its very high resolution, it has not been applied extensively to study biological or pathology samples. Here we report the application of this powerful technology to examine the three-dimensional ultrastructural characteristics of proteinuric glomerulopathy in mice with CD2-associated protein (CD2AP) deficiency. HIM revealed the serial alteration of glomerular features including effacement and disorganization of the slit diaphragm, followed by foot process disappearance, flattening and fusion of major processes, and eventual transformation into a podocyte sheet as the disease progressed. The number and size of the filtration slit pores decreased. Strikingly, numerous “bleb” shaped microprojections were observed extending from podocyte processes and cell body, indicating significant membrane dynamics accompanying CD2AP deficiency. Visualizing the glomerular endothelium and podocyte-endothelium interface revealed the presence of endothelial damage, and disrupted podocyte and endothelial integrity in 6 week-old *Cd2ap*-KO mice. We used the HIM technology to investigate at nanometer scale resolution the ultrastructural alterations of the glomerular filtration apparatus in mice lacking the critical slit diaphragm-associated protein CD2AP, highlighting the great potential of HIM to provide new insights into the biology and (patho)physiology of glomerular diseases.

## Introduction

One major function of the kidney is ultrafiltration of plasma via its glomerular filtration barrier that consists of podocytes, endothelial cells, glomerular basement membrane (GBM)^1^. The major and critical component of the glomerular filtration barrier is the podocyte, a specialized cell composed of cell body, major processes and foot processes^2^. Foot processes from adjacent podocytes interdigitate and form a specialized intercellular adhesion structure, the slit diaphragm (SD). Together with GBM and possibly other components, SD contributes importantly to the permeability of glomerular filtration barrier^3^. The SD is a highly organized and dynamic structure. It contains a complex of proteins including the podocin, nephrin, CD2-associated protein (CD2AP), and other SD-associated proteins such as zona occuldens-1 (ZO-1)^1^. Disruption of any components of the SD leads to glomerulopathy that is frequently manifested by proteinuria^1^.

CD2AP was identified originally as an adapter protein that interacts with the cytoplasmic domain of CD2, a membrane protein in T cells and natural killer cells^4^. In the kidneys, CD2AP is expressed at the cytoplasmic face of the SD and anchors podocin and nephrin to the actin cytoskeleton in podocytes^5, 6^. CD2AP deficient mice developed significant proteinuria with extensive foot process effacement and died in 6–7 weeks after birth^7^. *Cd2ap-knockout* (KO) mice provide a good model of proteinuric glomerulopathy due to SD protein deficiency.

For decades, scanning electron microscopy (SEM) has been commonly used to examine the complex, three-dimensional structure of the glomerular filtration barrier in the normal kidney and under various pathological conditions in experimental animals and in patients with various glomerular diseases^8–11^. Conventional SEM has enabled us to image podocyte ultrastructural features, including podocyte cell body, major processes and interdigitation formed by adjacent foot processes, and detect the effaced foot process in proteinuric glomerulopathy^12–14^. Continuous improvement of conventional SEM technology over several decades has allowed visualization of many important ultrastructural features of the normal and diseased glomeruli and tremendously advanced our understanding of the biology and pathophysiology of the kidney glomeruli. For example, more recently, the serial block-face SEM has been used to characterize normal glomerular structure and glomerulopathy using a series of ultrastructural sectional images. This method provides a better resolution for imaging glomerular structures. In addition, it revealed a novel ridge-like prominence connecting the foot processes which has not been observed by conventional SEM or any other electron microscopy techniques before^15^. Although the method is promising, it is very labor intensive and still limited by the resolution capacity that SEM is able to offer.

With the rapid advance of cellular and molecular biology and biotechnology in general, there is an increasing demand for the development of powerful microscopic technologies able to detect and/or characterize sophisticated molecular and cellular events occurring on the cell surface, and to rediscover fine structural features at nanometer resolution scale. SEM imaging is limited by its resolution at high magnification, by sample charging, and by the necessity of conductive coating, which obscures the fine structural details of the sample^16^. Here we report the application of a novel scanning microscopy technology, the Helium Ion microscopy (HIM) in proteinuric glomerulopathy due to the deficiency of a SD protein, CD2AP.

Unlike conventional SEM, HIM uses a scanning beam of He^+^ ions. One of the critical technological advances in HIM is the remarkably reliable and stable ion source, in which the tip is shaped so that its apex only consists of three atoms^16, 17^. The gun is centered such that only one of the three atoms is selected as the ion source and used for imaging^17^. Because of the extreme high brightness of the gas field ionization source, a ultra-small aperture can be used to define the He^+^ beam, therefore reducing chromatic and spherical aberrations of the optical column^16^. Consequently, due to the very small spot size and convergence angle on the imaged sample, HIM can provide high-resolution images with a much larger depth of field with no loss of surface detail. Another advantage over SEM is the ability of HIM to image uncoated biological samples. Sample charging severely affects image quality in SEM when imaging uncoated samples, therefore requiring metal coating, which could potentially be a cause of artifacts due to masking or obscuring details of the surface topography. In addition, HIM offers more access to deeper regions of the sample. The higher mass of He^+^ leads to a strong forward scattering of these ions and thus to their deeper penetration in the sample^16^. The deeper penetration of the He^+^ ions and the lack of energy deposition on the sample surface also cause minimal surface damage^16^.

With these advantages, HIM has recently been used for characterizing various biological samples, including cancer cells, platelets and extracellular tooth enamel matrix which provided many interesting new findings^18–20^. For example, HIM images depicted cancer cell-platelet aggregates, revealing a complex and intricate network of cellular interactions based on the adhesion of platelet pseudopodia to the surface of cancer cells^19^. More recently, our group used HIM to examine the morphology of the normal rat kidney revealing the glomerular ultrastructure with unprecedented clarity and detail^21^. In the present study, we used the powerful HIM technology to re-examine the three-dimensional ultrastructure of the glomeruli in a proteinuric glomerulopathy mouse model with CD2AP deficiency. Our data revealed multiple ultrastructural abnormalities of the glomeruli in *Cd2ap*-KO mice, including the appearance of numerous “bleb” shaped microprojections, intercellular junctional structure in effaced foot processes and disrupted glomerular endothelium. More interestingly, a complex interface consisting of podocyte, endothelium and glomerular basement membrane was readily visualized by HIM. These exciting findings support the utility of this high power scanning microscopy technology in the comprehensive research of glomerular diseases.

## Results and Discussion

### Glomerulopathy and proteinuria in CD2AP deficient mice

**Figure 1:**
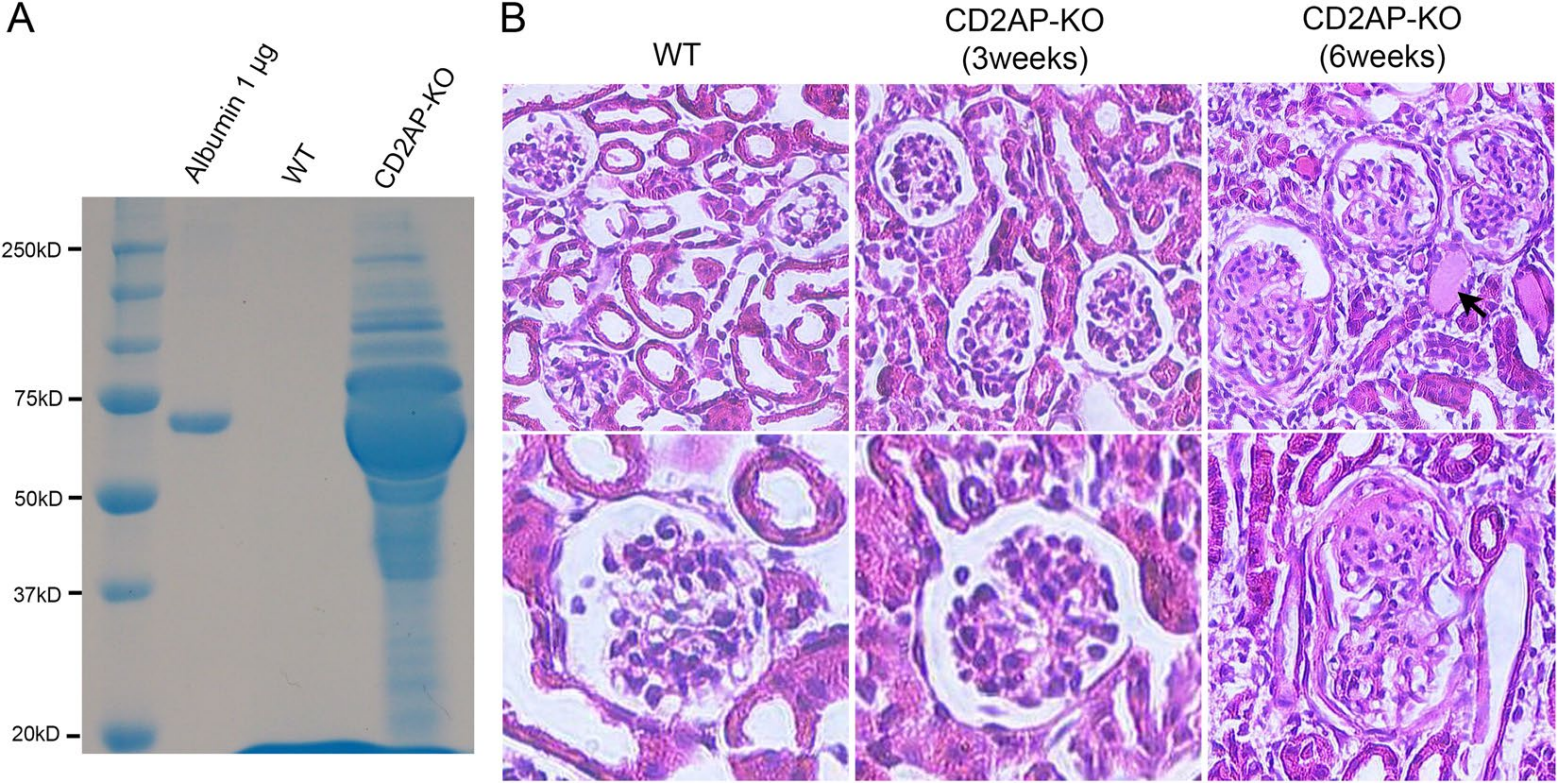
Analysis of proteinuria and kidney histology. (**A**) Coomassie blue staining reveals a significant amount of proteinuria of a wide molecular weight range in 6 week-old *Cd2ap-*KO mice but not in the WT mice. (**B**) Representative images of H&E staining in WT, 3 week- and 6 week-old *Cd2ap*-KO kidneys. While there are no obvious abnormalities of the glomerular structure in 3 week-old *Cd2ap*-KO kidneys compared to WT, nearly half of the glomeruli show segmental or global sclerosis. Moderate tubular atrophy and protein casts (arrow) were also detected in kidney tubules.

CD2AP deficient mice^7, 22^ were used at 3 and 6 weeks of age respectively. Spot urines from wild type (WT) and *Cd2ap*-KO mice were collected to test for proteinuria by SDS-PAGE with Coomassie blue staining. A significant amount of proteinuria was detected in all CD2AP deficient animals (Fig. 1A). H&E staining of the kidneys revealed no obvious abnormalities of the glomeruli in 3 week-old CD2AP deficient mice compared to WT (Fig. 1B), so these mice were considered to be at the early stage of the disease. In 6 week-old CD2AP deficient mice, nearly half of the glomeruli showed segmental or global sclerosis, and moderate tubular atrophy, Visible protein casts were detected in kidney tubules. These mice were considered to be at the late stage of glomerulopathy (Fig. 1B)^7^.

### Three-dimensional view of podocyte structure in CD2AP deficient mice

**Figure 2:**
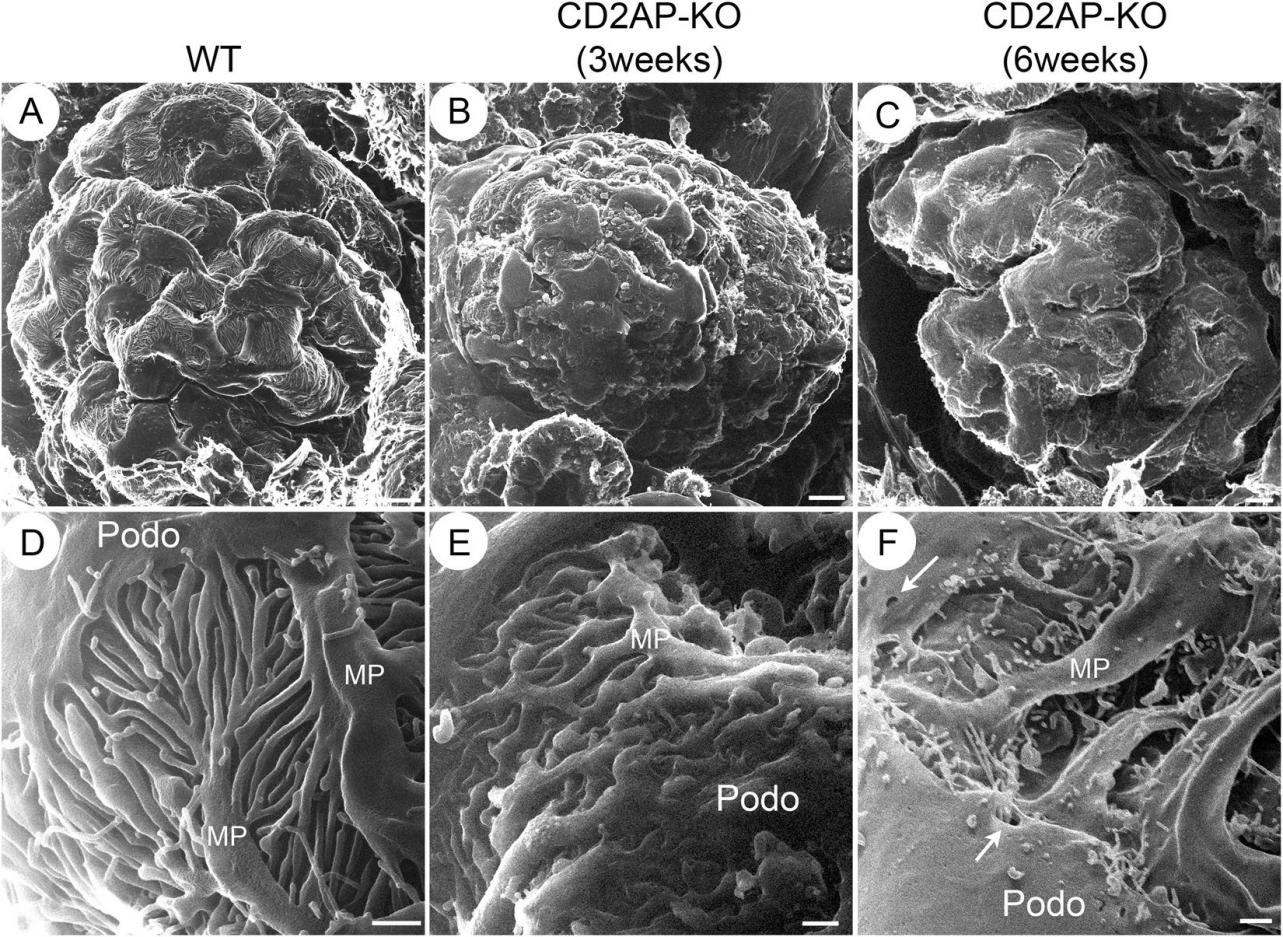
Glomerular and podocyte morphology in wild type (WT) and CD2AP deficient mice by HIM. HIM images of glomeruli in WT (**A**,**D**), 3 week-old *Cd2ap*-KO (**B**,**E**), and 6 week-old *Cd2ap*-KO mice (**C**,**F**). Low magnification image of a WT glomerulus (**A**) shows major processes and foot processes covering the glomerular capillaries, while images of 3 week-old *Cd2ap-*KO (**B**) and 6 week-old *Cd2ap*-KO kidneys (**C**) show a less regular and less smooth surface of podocytes. High magnification images of WT glomeruli (**D**) show a smooth podocyte cell body and well-organized interdigitating foot processes. A few filamentous microprojections originate from the podocyte cell body, major processes and foot processes. Conversely, the foot processes are swollen and appear short and severely flattened in the 3 week-old *Cd2ap*-KO kidney (**E**). Foot processes are fused and tend to disappear in 6 week-old *Cd2ap*-KO kidney (**F**). The primary processes are preserved, but appear “peeled away” from the underlying capillary loops. Numerous “bleb-shaped” short microprojections mixed with a few filamentous “cilia-like” structures project from the podocyte cell body. There are holes ranging from 100–400 nm in diameter on the surface of the podocyte cell body. Scale bars, 5 μm in upper panels; 500 μm in lower panels. Podo, podocyte; MP, major process.

HIM examination of the WT glomeruli showed that podocytes are well organized with branched major processes and interdigitated foot processes covering the glomerular capillaries. The smooth podocyte cell body and well-organized foot processes were clearly visualized at high magnification (Fig. 2, A and D). A few microprojections of different lengths protruded from the cell body, major processes and foot processes of the podocyte. The width of these microprojections averaged 57.6 nm, which was consistent with a previous report on rat kidney^21^. Multiple global abnormalities in CD2AP deficient mice were visualized by HIM. The CD2AP deficient podocytes were “flattened” or swollen with an irregular and rough/wrinkled cell surface in both 3 week- and 6 week-old *Cd2ap*-KO mice (Fig. 2-B, C, E and F). Foot processes were swollen and appeared short and severely flattened in the 3 week-old KO mice, and they were further effaced and became virtually absent in 6 week-old *Cd2ap*-KO mice (Fig. 2E and F, respectively). The primary processes were preserved but appeared “peeled away” from the underlying capillary loops in the late stage KO animals (Fig. 2F). Strikingly, numerous bleb-shaped structures mixed with a few filamentous microprojections protruded from processes and podocyte cell bodies in the KO animals (Fig. 2F). These microprojections clustered in the region of cell-cell contact and possibly indicated very active membrane dynamics at the podocyte periphery. Also interestingly, we observed some holes of various sizes ranging from 100 to 400 nm in diameter on the surface of the podocyte cell body in *Cd2ap*-KO but not in WT mice (Fig. 2F, arrows). We do not believe that this feature is an artifact due to sample preparation because we have never seen it in WT mice, nor in kidneys from a transgenic mouse model for a different glomerulopathy, the Alport syndrome mice (data not shown).

**Figure 3:**
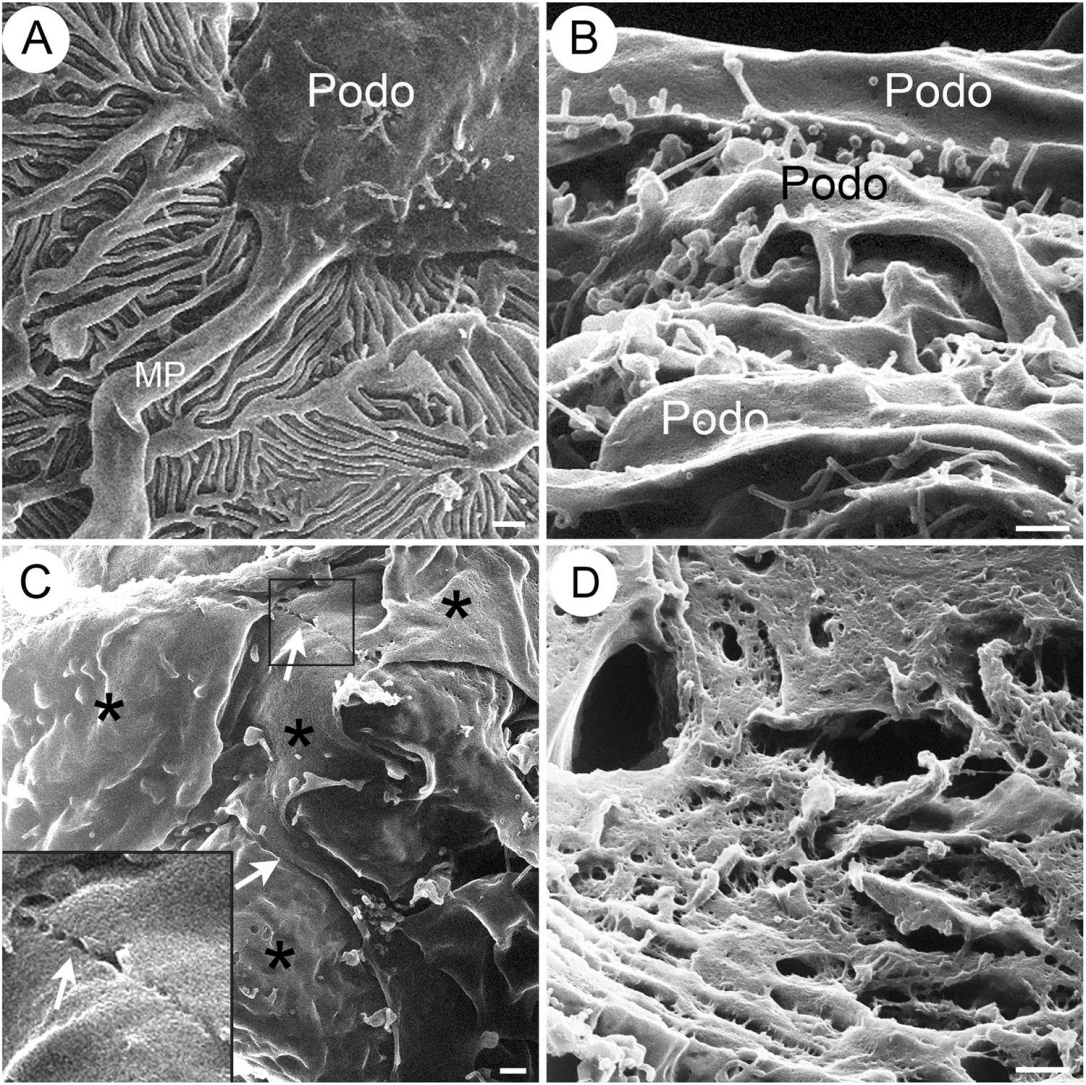
Podocyte ultrastructure at late stage in CD2AP deficient mice compared with WT mice. HIM images of podocytes in WT (**A**) and late stage *Cd2ap*-KO (**B**–**D**) mice are shown. (**A**) WT podocytes appear normal, with interdigitated structures formed by major processes and foot processes. Podocytes at late stage show broadly fused and flattened podocyte processes (**B**,**C**), and form irregular, flat sheets loosely covering the underlying structure (**C**, asterisk). Intercellular junctions (white arrows) are seen between some of these sheets (**C**). Terminally sclerosed glomeruli show uninterpretable glomerular ultrastructure (**D**). Some structures appear to be remnants of endothelial fenestrae or filtration slit pores that lost underlying membrane support. Scale bars, 500 μm. P, podocyte; MP, major process. Podo, podocyte; MP, major process.

Instead of the well-organized interdigitated foot processes seen in WT mice (Fig. 3A), we observed flattened podocyte processes in the late stage of glomerulopathy in *Cd2ap*-KO mice (Fig. 3B). Most podocyte processes had become completely effaced and formed an irregular, flat sheet that loosely covered the underlying structure (asterisks in Fig. 3C). Intercellular junctions were seen between some sheets (white arrows in Fig. 3C). At an even later stage, in terminally sclerosed glomeruli, glomerular ultrastructural features were uninterpretable. Some structures appeared to be remnants of endothelial fenestrae or filtration slit pores that had lost underlying membrane support, and merged with filamentous and bundled structures which are likely to be parts of the extracellular matrix (Fig. 3D).

**Figure 4:**
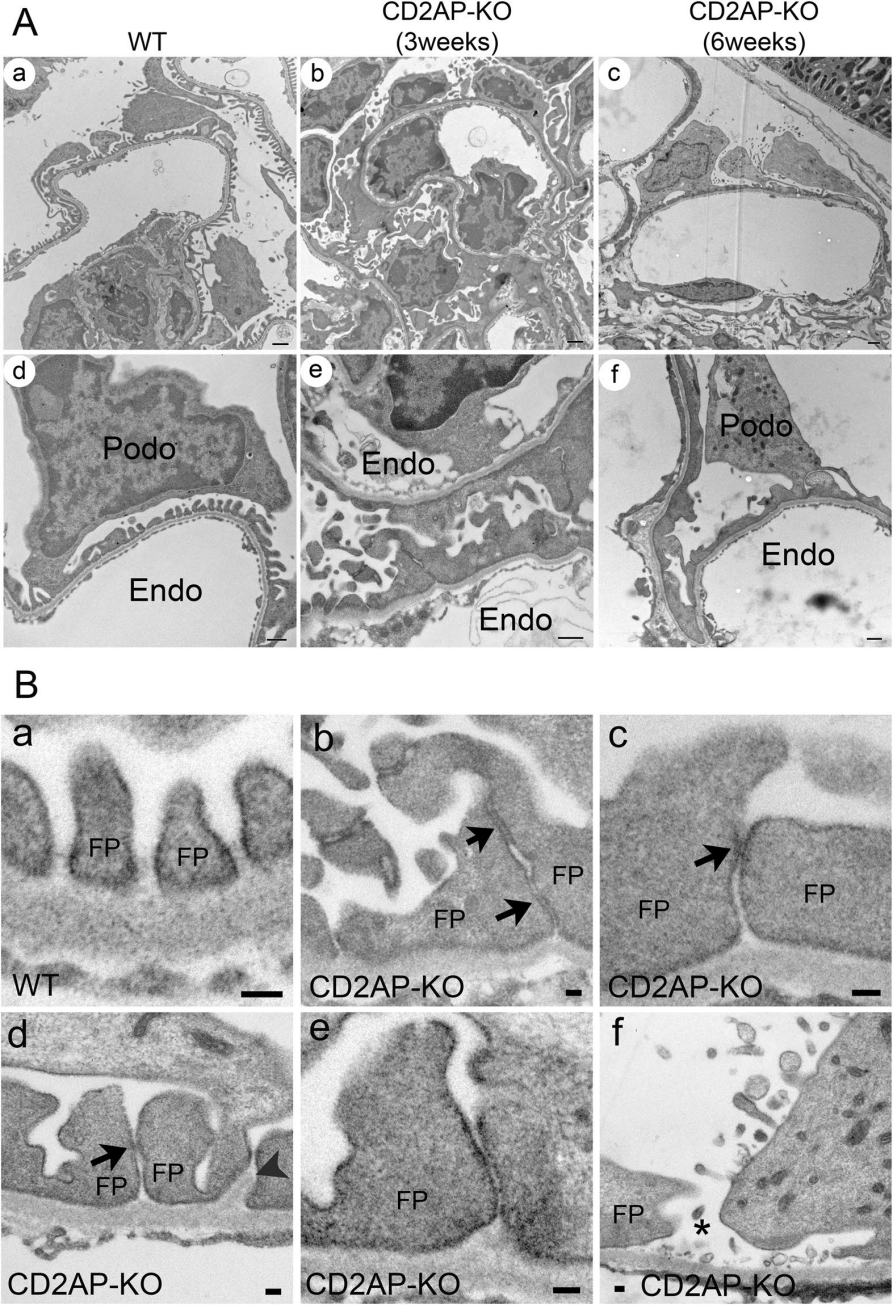
Podocyte morphology in WT and CD2AP deficient mice by TEM. (**A**) Representative TEM images of WT (a, d), 3 week-old *Cd2ap*-KO (b, e) and 6 week-old *Cd2ap*-KO (c, f) kidneys. WT glomeruli (a, d) show well-organized interdigitated foot processes lining up along the capillary wall with uniformly spaced slit diaphragms (SDs) between adjacent foot processes. Glomeruli of both 3 week- (b, e) and 6 week-old (c, f) *Cd2ap*-KO mice show massive foot process effacement, which is associated with the disappearance of the filtration slit and SDs between foot processes. Intracellular junction-like structures are frequently formed between effaced podocytes. Scale bars, 1 μm in upper panels; 500 nm in lower panels. (**B**) Representative TEM images of podocyte slit pores in WT (a) and *Cd2ap*-KO (b–f) mice. Intact slit pores are seen in WT mice (a). In *Cd2ap*-KO mice, narrow slit pores are frequently seen (b–e) with some cell contact area (black arrows). GBM expansion into the space between foot processes is observed (d, arrowheads). A widened slit pore with no SD is also observed (f, asterisk). Scale bar, 100 nm. Podo, podocyte; FP, foot process; Endo, endocapillary space.

**Figure 6:**
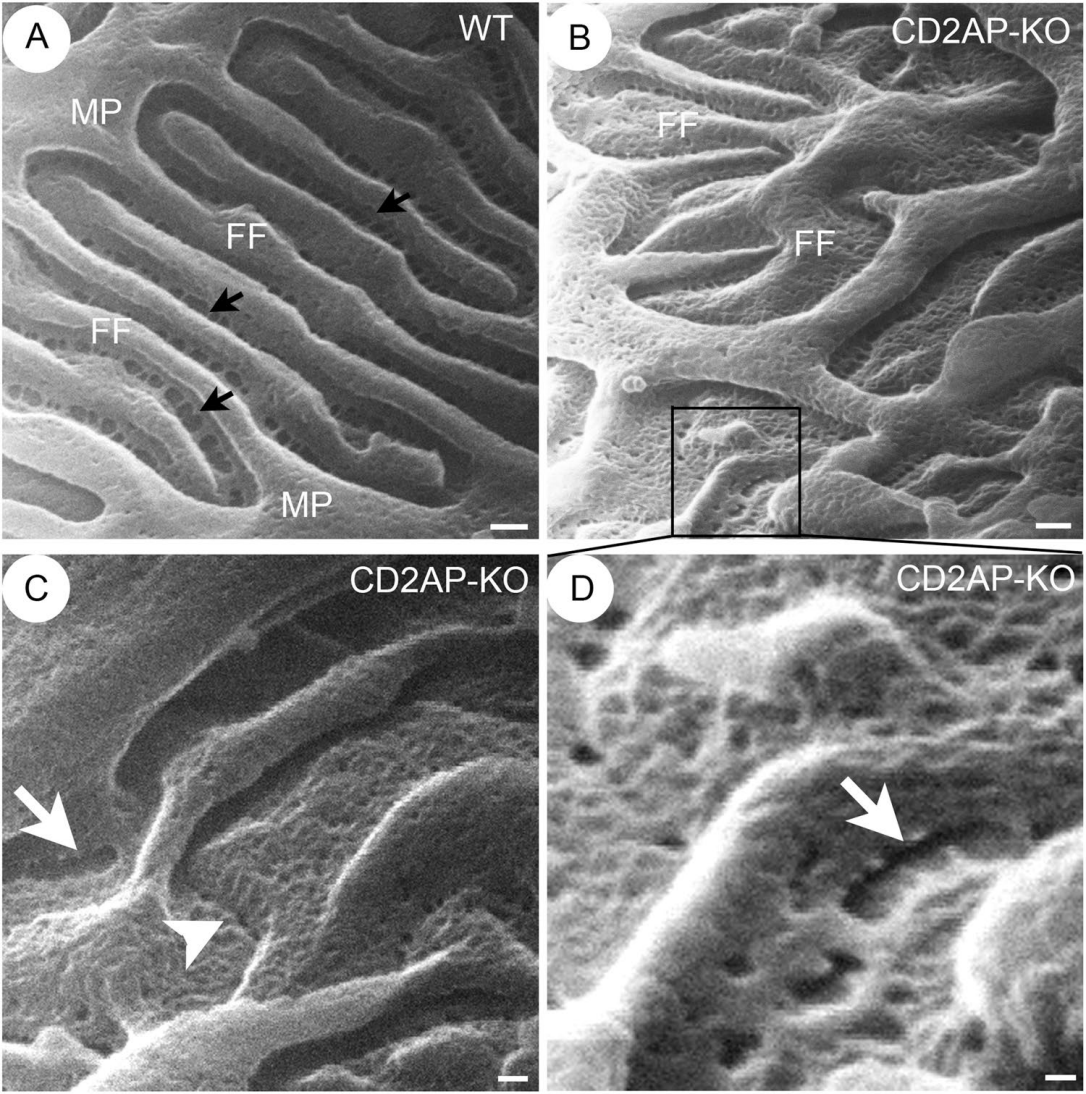
Foot process and filtration slit in WT and CD2AP deficient mice by HIM. HIM images of foot processes and SDs in WT (**A**) and 3 week-old *Cd2ap*-KO (**B**–**D**) kidney. Image of WT (**A**) shows interdigitated foot processes forming evenly spaced filtration silts. The slit pores (black arrows) line up and form a ladder-like pattern in the middle of the filtration slit and between adjacent foot processes. Image of *Cd2ap*-KO kidney (**B**) shows the disorganized and irregular orientation/alignment of primary process and foot processes. Foot process branching appears more random, and foot processes show flattened and widened (**B**). The “gaps” between disrupted SDs in some areas are detectable (white arrows, **C**,**D**). The junction between foot processes appears closed in some areas (white arrowhead, **C**). Scale bars, 100 nm (**A**,**B**); 50 nm (**C**,**D**). MP, major process; FF, foot process.

By transmission electron microscopy (TEM), WT glomeruli showed well-organized and interdigitated foot processes lined up along the capillary loops with uniformly spaced SDs in between adjacent foot processes (Fig. 4A-a,d). In both 3 week- and 6 week-old *Cd2ap*-KO mice, foot process effacement occurred and was associated with the disappearance of the filtration slit and SD between foot processes (Fig. 4A-b, c, e and f). The SD was replaced by tight-junction (TJ) structures that formed between processes and sometimes between cell bodies of adjacent podocytes (Fig. 4B). The SD to TJ transition has been frequently observed in many rodent models of proteinuric glomerulopathy, including protamine sulfate-treated animals, *NPHS1*-KO and *NPHS2*-KO, *ZO-1* KO and more recently *claudin-1* KO mice^7, 23–28^. This SD to TJ transition was also reported in nephrosis patients^29^. Interestingly, genetic disruption and alteration of the expression of TJ complex proteins was frequently associated with glomerulopathy. For example, the expression of ZO-1 is significantly decreased in the glomeruli from patients with diabetes mellitus and in the diabetes animal model *db*/*db* mice^27, 30, 31^. Podocyte-specific deletion of ZO-1 results in massive proteinuria^24^. Claudin-1, another protein that is primarily expressed in TJ of the glomerular parietal epithelium, is markedly upregulated in podocytes from *db*/*db* mice^32^. More recently, induction of Claudin-1 expression in podocytes was found to cause profound proteinuria in mice^23^. Taken together, these data suggest a more complex role of TJ in the pathogenesis of proteinuric glomerulopathy. Whether the SD-TJ transition contributes to the development of proteinuria is under active investigation. Nevertheless, using the HIM technology, we were able to detect TJ-like structures between adjacent podocytes that have never been clearly observed before, using the conventional SEM (Fig. 6C, arrowhead).

### Microprojections from podocytes in CD2AP deficient mice

**Figure 5:**
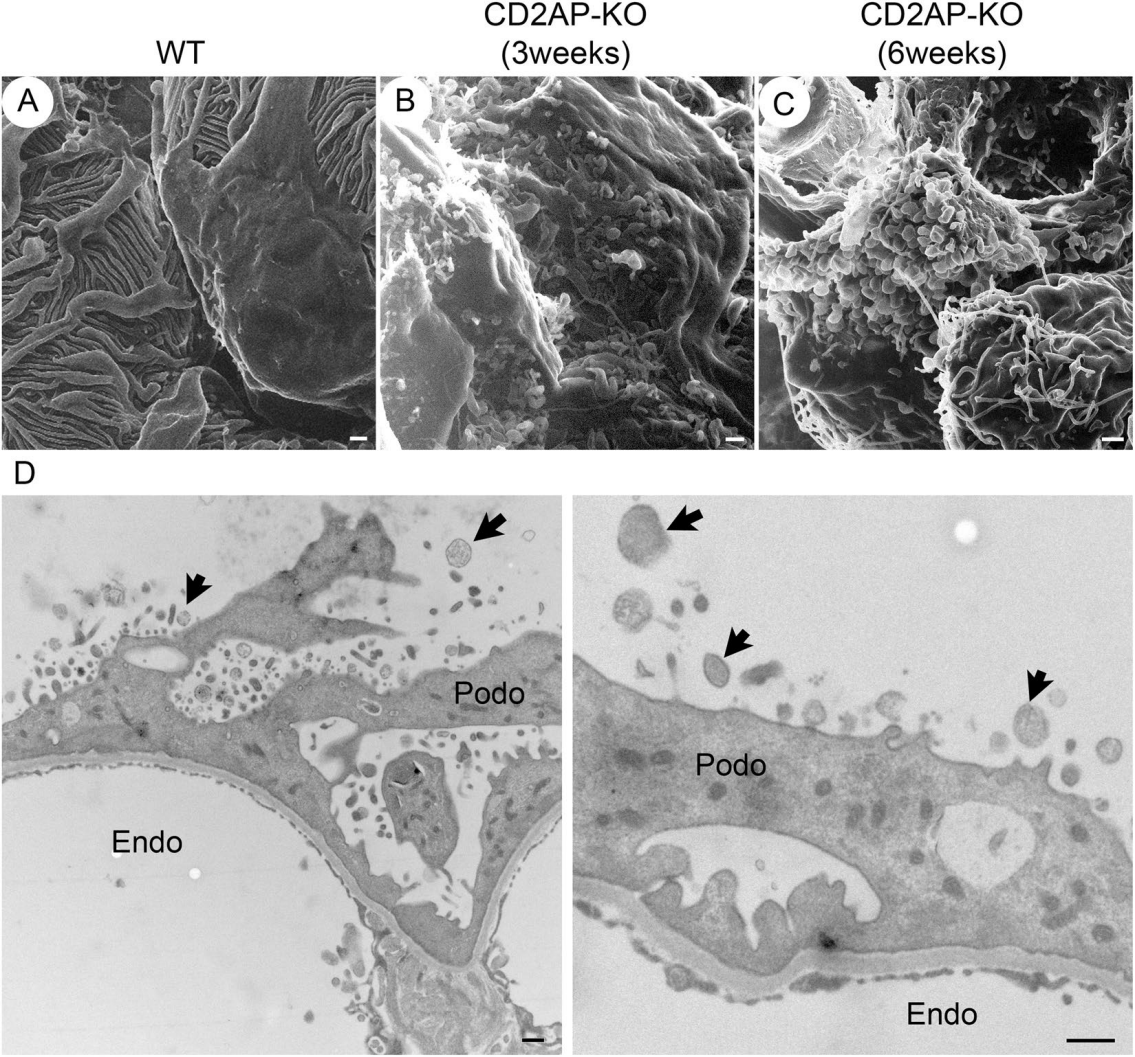
Bleb-like microprojections from podocytes in CD2AP deficient mice. HIM images of podocyte cell body in WT (**A**), 3 week-old *Cd2ap*-KO (**B**), and 6 week-old *Cd2ap*-KO mice (**C**). The image of WT podocytes (**A**) shows a smooth podocyte cell body and well-organized interdigitating foot processes. Images of 3 and 6 week-old *Cd2ap*-KO podocytes (**B**,**C**) reveal numerous “bleb-shaped” microprojections with a diameter of 200–700 nm on the surface of the podocyte cell body. Most of these blebs have a narrow “neck” connected to the cell body. Scale bars, 500 μm in upper panels. (**D**) TEM images of 6 week-old *Cd2ap*-KO glomeruli show numerous vesicle-like structures. Some of these structures have a narrow “neck” connected to the podocyte cell body. Scale bar, 500 nm. Podo, podocyte; Endo, endocapillary space.

One striking finding by HIM analysis was the appearance of numerous “bleb-shaped” microprojections in CD2AP deficient mice (Fig. 5B and C), while we could not detect any similar structures in WT mice (Fig. 5A). These microprojections were about 200–700 nm in diameter, and they all projected from the surface of the podocyte cell body in CD2AP deficient mice (Fig. 5C). Most “blebs” had a narrow “neck” connecting them to the podocyte cell body. These projections were also seen in *Cd2ap*-KO mice at 3 weeks of age (Fig. 5B). There were almost no detectable blebs or vesicle-like structures in WT glomeruli by TEM (data not shown). In contrast, there were numerous vesicle-like structures that appeared to be “secreted” by podocytes in *Cd2ap*-KO mice (arrows in Fig. 5D). The size, density and shape of these “vesicles” varied. They measured 100–700 nm in diameter, and had various shapes: spherical, tubular or irregular. The large variation in size, morphology and density of these vesicles (by TEM) and blebs (by HIM) illustrates the degree of heterogeneity of these membrane structures. As such, they may contain a mixed population of exocytotic vesicles such as microparticles and exosomes, as well as yet unidentified nanopods or nanotubules that are only minimally studied in bacteria^33, 34^.

Are these “blebs” reflecting a robust exocytotic activity on the podocyte surface or transcytosis from the podocyte membrane facing the GBM to the membrane facing the urinary space? Indeed, a new transcytotic pathway for protein transport was recently identified by intravital imaging studies^35^. Although it only accounts for 10% of total albumin filtered under normal conditions, this transcytotic pathway could be greatly augmented by disease conditions such as CD2AP deficiency in our case. The numerous bleb-shaped microprojections may indicate an intensified exocytotic activity or abnormal membrane dynamics of podocytes that associates with massive proteinuria in CD2AP deficiency. Alternatively, it is also possible that these “microprojections” or vesicles could mediate genetic and/or cellular communication between cells, which may be up-regulated by cellular stress in *Cd2ap*-KO glomeruli^36, 37^.

### Foot process and filtration slits in WT and CD2AP deficient glomeruli

High resolution HIM images showed that the interdigitated foot processes formed an evenly spaced filtration slit in the WT glomerulus (Fig. 6A). The filtration slit pores lined-up and formed a ladder-like pattern in the middle of the filtration slit and between two adjacent foot processes (Fig. 6A). In *Cd2ap*-KO mice, even at 3 weeks of age, the orientation and alignment of primary processes and foot processes were disorganized (Fig. 6B). Foot processes branched more randomly and appeared more flattened and wider compared to those in WT mice (Fig. 6B and C). The size of the slit pores was smaller (Fig. 6B–D), the number of SD pores was reduced, and foot processes were shorter (Table 1) in the *Cd2ap*-KO kidneys. Reductions in the number and size of slit pores were previously observed in proteinuric animals and in nephrosis patients^29, 38, 39^. It has been proposed that slit pore reduction and foot process effacement may lead to high resistance and increased perfusion pressure across the filtration barrier, eventually causing barrier failure^40^.

**Table 1:** Podocyte slit pores, slit gaps and length of foot processes in wild type (WT) and CD2AP deficient mice.

	WT	CD2AP-KO (3-week old)
*Slit pores*		
Number of pores/μm FP	27.44	20.68
*Slit gaps*		
Number of slit gaps/FP	0/101	4/60
Length of FP (nm)	1654 ± 79	460 ± 29

FP, foot process. The number of slit pores are analyzed in WT (7.47 μm of FP length) and 3 week-old *Cd2ap*-KO (11.94 μm of FP length) mice. We defined “slit gap” as occurring when three or more consecutive cross-bridging filaments separating slit pores are broken. Length of FP is measured in WT (n = 30) and 3 week-old *Cd2ap*-KO (n = 24) mice, and presented as means ± standard error of the mean, SEM.

Occasionally, we observed by HIM disrupted SDs that formed “gaps” in some areas in 3 week-old *Cd2ap*-KO mice (Fig. 6C and D), and very rarely, we also saw a “gap” between the FP of podocytes by TEM (Fig. 4B–f). The occurrence of large gaps in glomerular filtration slits is not surprising. Lack of SD proteins such as CD2AP and nephrin is known to cause disruption of the SD protein complex and of the SD, and associates with proteinuria in rodents and human patients^1^. Throughout this study we have not observed podocyte detachment from GBM or denuded GBM by either HIM or TEM that would contribute to protein leakiness. Similarly, podocyte detachment and GBM denudation have not been observed routinely in human kidney biopsies in proteinuric glomerular diseases.

With any sample preparation for imaging analysis, there is always a concern that the observed change in the size of the slit pore and the presence of gaps between adjacent foot processes could be artifacts occurring during sample preparation, possibly caused by the commonly high osmolality of the fixatives that are used. In this study, we used a modified paraformaldehyde-lysine-periodate (PLP) fixative^21^. The measured osmolality of this fixative is 935 mOsm/kg, which is considerably lower than the osmolality of several other widely used fixatives, such as Karnovsky’s fixative^41^. This modified PLP fixative has been used for over two decades in immunofluorescent staining, TEM and SEM studies performed by our group and other groups^42–46^. We have never noticed evidence of significant cell shrinkage or dilation of intercellular spaces induced by this fixative. In addition, the series of abnormalities observed in *Cd2ap*-KO mice were not seen in WT mice, which argues further against the possibility of an artifact.

Using these high-resolution images, we were able to reliably measure the size and area of the SD. In WT kidneys, the average width of the SD was 43.6 ± 0.4 nm (mean ± standard error of the mean, SEM, n = 130), and the average width of the slit pore was 26.5 ± 0.4 nm (mean ± SEM, n = 124). The average area of the slit pore was calculated as 730 ± 26 nm^2^ (mean ± SEM, n = 80). Rice *et al*.^21^ reported that the mean width of the slit pore is 22.0 nm in normal rat kidney imaged by HIM, which is very consistent with our measurement. On the other hand, Gagliardini *et al*.^47^ previously reported using SEM of metal-coated samples that the average SD pore radius is 12.1 nm (corresponding to a 24.2 nm diameter, i.e. width of SD pore) and the mean pore area is 494 nm^2^ in Wistar rats, whereas mean pore area in Munich Wistar Frömter (WMF) rats is 564 nm^2^. The discrepancy in the size of the slit pore is not large despite two very different procedures/technologies. It is conceivable that sample coating with heavy metals partially covers the pore edges, such that pore size appears smaller by conventional SEM. It is also possible that the variation in the size of the slit pore is due to the different preparation procedures applied. Gagliardini *et al*. used hexamethyldisilazane (HMDS) to dehydrate the kidney samples, whereas our current study and Rice *et al*. used critical point drying (CPD). Interestingly, Gagliardini *et al*. reported that the filtration slit shows the same heteroporous appearance when kidney samples are dehydrated with HMDS or by CPD, implicating no detectable difference in these two preparation procedures in their hands. Even though we cannot completely rule out that the sample preparation used in our study affects the pore structure, the same preparation protocol in previous studies by our group was able to effectively preserve very fragile structures, such as the central cilium in kidney collecting duct principal cells, microplicae with deep infoldings in the apical membranes of collecting duct intercalated cells^21^, similarly complex microplicae after cAMP treatment in epididymal clear cells, and stereocilia of epididymal principal cells^48^.

Another point of contention is the structure and architecture of the SD. Our images show the presence of a ladder-like pattern in the middle of the filtration slit between two adjacent foot processes, as previously reported^21, 47, 49^. Our measurements indicate that the cross-bridging filaments that separate the slit pores have an average length of 43.6 nm. In a recent study, Grahammer *et al*., using plastic electron tomography, found a population of strands, attributed to nephrin, with a mean length of 45 nm joining neighboring foot processes towards their apical side^50^. It is possible that the cross-bridging filaments we observed are the same or similar structures as the nephrin strands described by Grahammer *et al*.

### Alteration of glomerular endothelium and podocyte-endothelial interface in the CD2AP deficient glomeruli

**Figure 7:**
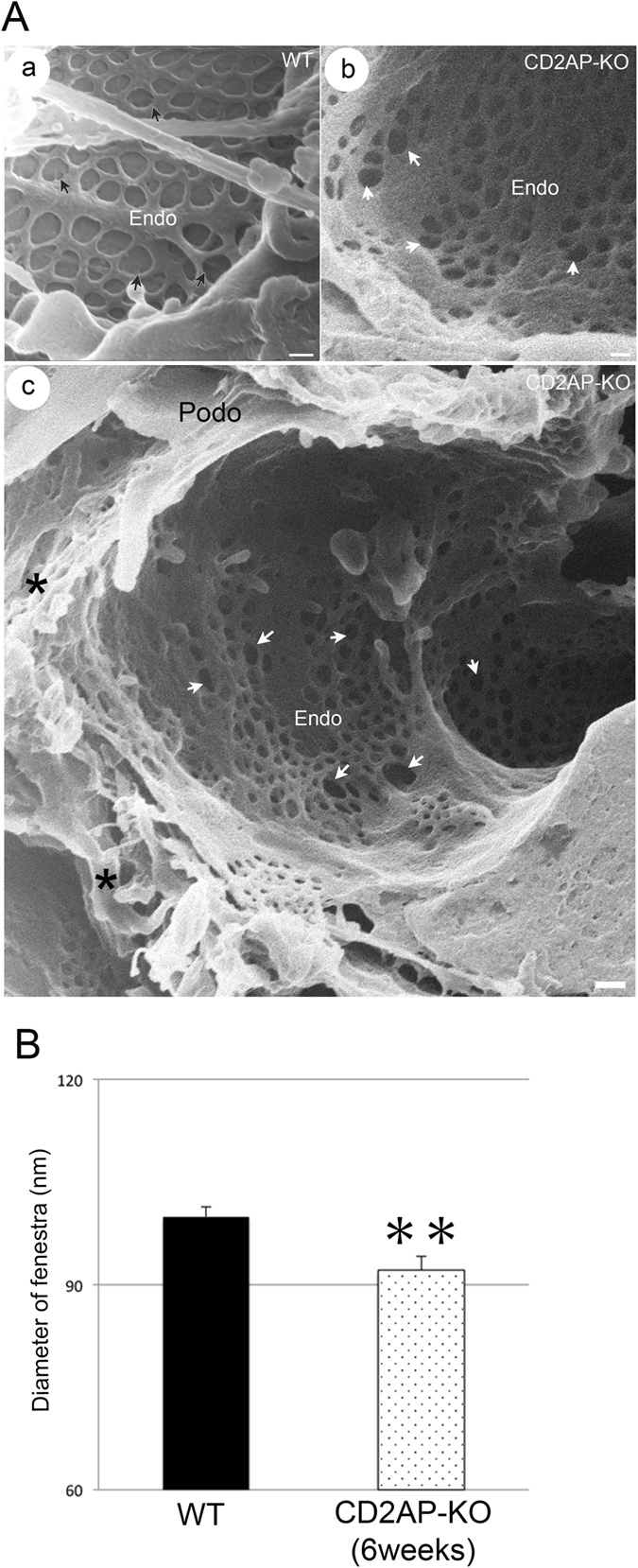
Endothelium and interface of podocyte and endothelium in WT and CD2AP deficient mice. (**A**) HIM images of glomerular filtration slits in WT (a) and 6 week-old *Cd2ap*-KO (b, c) mice. (a) The WT image shows the smooth endothelial surface and endothelial fenestrae with membranous structure underneath. Some of these fenestrae contain faint spokes that appear like bicycle wheels (black arrows). (b) The image of 6 week-old *Cd2ap*-KO endothelium shows smaller fenestrae, many of which lost underlying membrane structure, revealing many open “holes” underneath (white arrows). Scale bars, 100 nm. (c) HIM image of the interface between podocytes and endothelium in 6 week-old *Cd2ap*-KO mice. *Cd2ap*-KO mice lost interdigitated foot processes. There are multiple layers of overlaid “flat sheets” that loosely attached to the underlying GBM and endothelium (asterisk). The inner endothelial surface contains numerous fenestrae, most of which lack underlying supporting structures (white arrows). Scale bars, 100 nm (a, b); 200 nm (c). Podo, podocyte; Endo, endocapillary space. (**B**) The diameter of randomly selected endothelial fenestrae is quantified by ImageJ software in WT (n = 46) and 6 week-old *Cd2ap*-KO (n = 78) from HIM images. All values are presented as means ± standard error of the mean, SEM. **p < 0.01.

Examination by conventional SEM of the ultrastructural abnormalities of the glomerular endothelium has been limited, although TEM data suggest some endothelial damage in various glomerulopathies^51–53^. By HIM, we observed numerous endothelial fenestrae in WT mice, of an average diameter of 100 nm (Fig. 7A–a). There was a membranous sheet-like structure underneath endothelial fenestrae (Fig. 7A-a). In the 6 week-old *Cd2ap*-KO mice, the endothelium appeared thicker and endothelial fenestrae were significantly smaller (Fig. 7B). Interestingly, many endothelial fenestrae lost the underlying membranous structure, leaving open “holes” underneath, which indicates a disintegration of the underlying GBM (white arrows in Fig. 7A-b).

Another important advantage of HIM is that it enables a direct visualization of the podocyte-endothelium interface, which has not been achieved in the past with conventional SEM (Fig. 7A-c). The transverse view of glomeruli from 6 week-old *Cd2ap*-KO mice revealed the loss of normal interdigitated foot processes. There were multiple layers of overlaid “flat sheets” that loosely attached to the underlying GBM and endothelium (asterisks in Fig. 7A-c). The inner endothelial surface contained many endothelial fenestrae, many of which lost the underlying supporting membranous structure, probably the GBM (white arrows in Fig. 7A-c). The cross talk between podocyte, endothelial cell and GBM is critically important but still poorly understood. Glomerular endothelial cells and podocytes both lie on the GBM where cross talk is thought to occur through diffusion of cytokines/growth factors^54^. Many studies, especially genetic studies in animals have suggested that podocytes may communicate with endothelial cells through secretion of multiple paracrine factors including vascular endothelial growth factor-A (VEGF-A), angiopoietin-1 (Ang-1), angiopoietin-2 (Ang-2), endothelin-1 (ET-1), angiopoietin like 4 (ANGPTL4), stromal cell-derived factor 1 (SDF-1) and interleukin 6 (IL-6) while endothelial cells interact with podocytes by secreting factors such as hepatocyte growth factor (HGF), insulin-like growth factor (IGF) and tumor necrosis factor-α (TNF-α)^54^. For example, Gnudi *et al*. reported that mice with podocyte-specific Ang-1 overexpression in diabetic kidney diseases prevented diabetes-induced glomerular endothelial cell proliferation and reduced albuminuria^55^. On the other hand, Siddiqi *et al*. reported that diabetic mice with endothelial dysfunction induced by genetic deficiency of the endothelial nitric oxide (NO) synthase, developed a podocyte-specific injury and heavy albuminuria, indicating a detrimental impact of endothelial cell dysfunction on podocytes^56^. Therefore, it is believed that a proper and comprehensive cross talk between endothelial cell and podocyte is essential for the normal function and structure of the glomerular filtration barrier. Despite the fact that endothelial damage has not been reported in glomerulopathy associated with SD defects, such as CD2AP deficiency in the past^7, 22^, we are now able to directly observe the endothelial abnormalities resulted from the CD2AP deficiency in mice through HIM. Further comprehensive studies using HIM are needed to advance our understanding of the interplay of signals between podocyte, endothelium and GBM.

In comparison with conventional SEM, HIM is able to reveal much more ultrastructural details with higher resolution. For example, a previous SEM study of nephrin-deficient glomeruli only revealed the presence of prominent podocyte cell body, abundant microvillar structures, long primary processes and foot process effacement^57^. In our HIM study of the *Cd2ap*-KO kidney, we clearly observed evolutional alteration of glomerular structures starting with effacement and disorganization of the SD, followed by loss of podocyte foot processes, flattening and fusion of podocyte major processes, and eventually their transformation into a flat sheet. The presence of numerous “bleb” shaped microprojections protruding from podocyte processes and cell body, suggests significant membrane dynamics that was associated with the CD2AP deficiency. In addition, disruption of endothelial integrity at the podocyte-endothelium interface was clearly visualized by HIM. Taken together, HIM results have evidently offered an important advantage over SEM due to its nanometer scale resolution. The implications of the understanding and (re-) discovering of glomerulopathy-associated ultrastructural abnormalities for a specific glomerular pathophysiology are, however, difficult to anticipate completely. The incomparable structural details and the nanometer scale resolution indicate an enormous potential for applying this powerful technology to study the very complex filtration barrier structure and function, and thus for shedding light on the specific underlying pathophysiology of various glomerular diseases.

## Materials and Methods

### Animal experiments

Animal experiments were approved by the Washington University Animal Studies Committee, in accordance with the National Institutes of Health Guide for the Care and Use of Laboratory Animals. 2–3 month-old male mice (C57BL/6 J) were used as wild type controls. *Cd2ap*-KO mice (3 week-old and 6 week-old) were generated on the C57BL/6 J background as previously described^58^. All mice were anesthetized by intraperitoneal injection of pentobarbital sodium (60 mg/kg body weight, Nembutal, Abbott Laboratories, Abbott Park, IL) and perfused through the left cardiac ventricle with phosphate-buffered saline (PBS, 0.9% NaCl in 10 mM phosphate buffer, pH 7.4) at the speed of 10–15 ml/min for 5 min, followed by modified paraformaldehyde-lysine-periodate (PLP) fixative: paraformaldehyde (4%), lysine (75 mM), sodium periodate (10 mM) and 0.15 M sucrose in 37.5 mM sodium phosphate for 5 min^21^. These tissues were subjected to post-fixation with modified PLP at 4 °C overnight, followed by washing with PBS. For TEM and HIM analysis, tissues were treated with additional fixation with 2% glutaraldehyde (GA) in 0.1 M sodium cacodylate buffer, pH 7.4 (Electron Microscopy Sciences, Hatfield, PA), followed by washing with 0.1 M sodium cacodylate buffer and then PBS. All the tissues were stored in PBS containing 0.02% NaN_3_ at 4 °C until processing for the critical point drying process. Spot urine from each mouse was collected for the analysis of proteinuria at the time of sacrifice.

### H&E staining and urinary analysis

Modified PLP-fixed kidney tissues were embedded into paraffin and sectioned at 5-µm thickness. Sections were stained with hematoxylin and eosin (H&E). For analysis of proteinuria, spot urine samples (2 µl) collected from each mouse at the time of sacrifice were mixed with SDS-sample loading buffer and subjected to 10% SDS-PAGE. Gels were stained with SimplyBlue^TM^ SafeStain (Invitrogen, Carlsbad, CA) for 1 hour and then washed with double-distilled water (ddH_2_O) for 1 hour. Bovine serum albumin (Santa Cruz Biotechnology, Dallas, TX) was used for the albumin control band.

### Alcohol replacement and critical point drying (CPD)

Thin kidney slices (~500 μm) were exposed to a series of graded methanol solutions with the following alcohol dilutions and schedule: 25% methanol in PBS for 60 min, 40% methanol in PBS for 45 min, 60% methanol in ddH_2_O for 45 min, 80% methanol in ddH_2_O for 45 min, all at room temperature and then 80% methanol in ddH_2_O overnight at 4 °C, followed by 100% methanol for 60 min^21, 48^. For each grade, the alcohol solution was refreshed halfway through the incubation. Dehydrated kidney slices were then subjected to CPD in metal baskets using a Samdri-795 apparatus (Tousimis Research Corporation, Rockville, MD) as previously described^48^. Tissues were maintained at supercritical parameters (850–900 psi, >42 °C) for 4 min and the pressure was then reduced at a rate of <100 psi/min.

### Helium ion scanning microscopy (HIM)

HIM was performed as previously described^21, 48^. Dried kidney samples were loaded into the Orion helium ion microscope (Carl Zeiss Microscopy, Peabody, MA) and were maintained at a pressure of 2–3 × 10^−7^ torr throughout the analysis. Images were obtained by collecting the secondary electrons resulting from the interaction of the sample with the helium ion beam, using an Everhart-Thornley Microchannel plate. The signal was amplified by a photomultiplier tube, digitized with an A/D converter, and displayed as a grey value in an image pixel. The signal was acquired in line-averaging mode, with 64 lines for each line of the final image. An electron flood gun was used for charge neutralization after each line pass.

### Transmission electron microscopy (TEM)

Modified PLP-fixed kidneys were additionally post-fixed for 1 hour at room temperature with 1% osmium tetroxide in cacodylate buffer and were exposed to a graded series of ethanol solutions to 100% for dehydration. They were then infiltrated with Epon resin (Ted Pella, Redding, CA), sectioned, and examined in a JEOL JEM 1011 transmission electron microscope (JEOL, Peabody, MA) as previously described^59^. Images were collected using an AMT digital imaging system (Advanced Microscopy Techniques, Danvers, MA).

### Data analysis and statistics

Statistical analysis was carried out according to Handbook of Biological Statistics by Dr. John H McDonald, University of Delaware (http://www.biostathandbook.com/index.htm). A Student *t*-test was used to evaluate the difference between individual groups and a *P* value of <0.05 was considered to be significant. Data is reported as mean ± SEM, and error bars indicate SEM.
